# Optimization and selection of cause marketing mode with the warm glow effect

**DOI:** 10.1371/journal.pone.0272724

**Published:** 2022-08-11

**Authors:** Chuanliang Wu, Jiaping Xie, Tingting Zhang

**Affiliations:** 1 College of Business, Shanghai University of Finance and Economics, Shanghai, PR China; 2 College of Economics and Management, Huainan Normal University, Huainan, P. R. China; University of Wolverhampton, UNITED KINGDOM

## Abstract

Cause marketing (CM) is an important way of implementing corporate social responsibility (CSR) strategies. While most related studies explore firms’ implementation of CM campaigns, which involve donation of part of their sales revenue to charity for a social cause, we focus on the case of a firm contributing a specific ratio of its sales quantity to implement the CM campaign and divide the CM campaign mix into four modes according to different CM implementation subjects and the wholesale price (exogenous or endogenous). Unlike firms in the supply chain that use donation amounts to implement CM, the implementation of CM by donation ratio will be influenced by the donation cost, which can further affect their pricing strategies. Therefore, this study takes a two-level supply chain as the research object and builds Stackelberg game models to explore the optimization problem of donation and pricing decisions for different CM modes and choices from CM modes. This study presents three main conclusions. First, when the degree of preference for CM is sufficiently large, the supplier or retailer can implement CM only when the income generated by the increase in sales and retail price can compensate for the donation cost. Owing to the differing donation costs, it is easier for suppliers to implement CM than retailers. Second, in the case of the exogenous wholesale price, when the degree of preference for CM is relatively low, the supplier should implement the CM. However, when the degree of preference for CM is relatively high, the retailer should implement the CM. When the degree of preference for CM is moderate, the supplier can suppress the free-rider behavior of the retailer in implementing CM by sharing donation costs with the retailer, thereby achieving a win-win situation. Third, in the case of endogenous wholesale prices, the supplier should take the initiative to implement CM. Compared with other CM modes, the donation ratio is the largest in this mode.

## 1. Introduction

In recent years, an increasing number of firms have focused on corporate social responsibility (CSR). For example, only 26 Chinese A-share listed firms issued CSR reports in 2007; this increased to 930 in 2019. Cause marketing (CM) is a meaningful way of realizing CSR. It improves social welfare and increases profitability for dual purposes [1]. Additionally, consumers enjoy higher utility from purchasing a cause-linked product because of the warm glow effect of satisfaction generated by altruistic behavior [2]. Varadarajan and Menon [3] characterized CM as an offer from a firm to contribute a specified amount to a designated cause when customers engage in revenue-providing exchanges that satisfy organizational and individual objectives. “Mktforgood,” a CM advocacy organization, released a research report on China’s CM consumption. The report shows that, in 2021, 89% of the respondents had participated in a CM campaign more than once, and nearly 63% had participated in a CM campaign less than five times. As more consumers join CM campaigns, firms have launched different CM campaigns, such as the “grass planting Himalaya” CM campaign launched by Chando. Since the implementation of that project, Chando has created a domestic sales record for its main product and has donated 10 million yuan to the China Environmental Protection Foundation [4].

A CM campaign can be launched by either an upstream supplier (e.g., Chando) or a downstream retailer. For example, Dick’s Sporting Goods ran a CM campaign in which, for every pair of athletic shoes sold, the firm donated $1 to PACE, a program that helps combat concussions in youth sports [5]. In addition, the wholesale price of cause-linked products may be endogenous or exogenous. For example, Chando’s CM campaign is a long-term marketing strategy, and the supplier can change the wholesale price according to the market situation. However, in most cases, we consider the wholesale price contract, as is commonly observed in practice; here, the wholesale price is considered exogenous [6, 7]. Therefore, the supply chain CM campaign can be divided into four modes according to different implementation subjects (supplier or retailer) and wholesale prices (exogenous or endogenous). What are the preconditions for firms to implement CM in different modes, the differences between them, and the factors affecting CM in the supply chain? These are practical problems of concern in academic and industrial circles.

The forms of donations for firms that implement CM differ. Additionally, in contrast to Chando’s sales revenue, the clothing brand Hodo implemented a CM campaign in the summer of 2018, donating one down jacket to children in poor areas for every ten down jackets sold [8]. TOMS shoes implemented the CM campaign of donating one pair for every pair sold in the initial marketing strategy of the company [9]. This type of CM campaign in which a specific ratio of sales quantity is donated has achieved great success. Gao [5] highlighted that the donation of sales revenue could not entirely reflect the actual utility of consumers for cause-linked products and may also be affected by retail prices. The ratio of donations is more suitable for firms to use to accurately obtain the utility changes of consumers during the CM implementation process. The total sales revenue or quantity of donations announced may be very high for large firms. However, the donation ratio relative to the total sales revenue or sales quantity is minimal. In this situation, the warm glow effect obtained by consumers when purchasing cause-linked products is determined by the total sales revenue or the quantity sold. This method is not conducive to the implementation of CM by small and micro firms. Therefore, our study assumes that firms implement CM by donating a specific ratio of sales quantity.

When the realization type of CM campaign was determined, we focused on the subject of CM implementation. The subjects of CM implementation can be either suppliers or retailers. Suppliers implementing CM have the advantage of being a supply chain leader in their own right. The wholesale price of short-term marketing activities is usually exogenous, and retailers implementing CM can optimize the decision variables from a centralized supply chain perspective. Thus, the question is who should implement CM or if a contract might be established to allow suppliers and retailers to jointly share donation costs. Therefore, optimization and selection of the CM mode has become an urgent problem for firms.

We constructed a supplier-led two-level supply chain CM Stackelberg game model and specifically studied the following issues: What are the preconditions and differences of CM in different modes? How does the degree of preference for CM affect the implementation of CM in the supply chain? Furthermore, how can we optimize and select the CM mode with the warm glow effect? What types of contracts can improve the implementation effect of CM?

The main research results are as follows. First, for firms to implement CM, they must have a sufficiently large degree of preference for CM, and the income brought by the increase in sales quantity and retail price must make up for the donation cost. In addition, it is easier for suppliers to implement CM because the donation cost of suppliers is lower than that of retailers. Second, in the case of the exogenous wholesale price, the supplier or retailer chooses whether to implement CM according to the different ranges of preference for CM. When the supplier or retailer wants the other to implement CM, the supplier should take the initiative to jointly bear the donation cost with the retailer to avoid the free-riding behavior of the retailer in implementing CM. Finally, the supplier should implement CM if the wholesale price is endogenous.

The remainder of this paper is organized as follows. Section 2 reviews the related literature. The model is described in Section 3. Sections 4 and 5 present the analysis of different CM modes in the case of exogenous and endogenous wholesale prices, respectively. Finally, conclusions and future research directions are presented in Section 6. All proofs are presented in the S1 Appendix.

## 2. Literature review

CSR has traditionally been conceptualized as a managerial obligation to protect and improve society’s welfare and the interests of organizations [10]. Tang [11] and Sodhi and Tang [12] highlighted eight areas of CSR that should receive attention and proposed six CSR research opportunities. Berengue et al. [13] and Besiou and Van Wassenhove [14] highlight CSR’s research potential and opportunities in the supply chain. There are many different types of marketing, such as green marketing [15, 16], advance selling [17] and digital marketing [18]. In this study, we research one type of CSR initiative, CM, and focus on the mode optimization and selection of firms implementing CM from the supply chain perspective.

Our study is related to the combination of CSR and supply chains. Nielsen et al. [19] generated a decision support framework from the perspective of the selection and successful implementation of environmentally friendly products by comparing the profits of each member, greening level, and so on. Pal et al. [20] and Xu et al. [21] analyzed a green supply chain system by considering many factors such as quality, and blockchain. Guo et al. [22] combined CSR with procurement and analyzed the procurement decisions of two types of suppliers from the supply chain perspective. Agrawal and Lee [23] studied the impact of a downstream buyer’s sourcing strategy on its suppliers’ CSR performance. Yu et al. [24] discussed whether government-funded projects should be biased towards consumers or manufacturers from the supply chain perspective for the policy benefit project of China’s “home appliances to the countryside” program. Moreover, Yu et al. [25] discussed the decision-making of firms and the government in the context of the residents’ need for solar lamps after the earthquake in Haiti, combined with the nature of the commercial and humanitarian supply chains, to determine who to subsidize (manufacturers, retailers, and consumers) and the degree of product subsidies. Meanwhile, different supply chain contracts and models may also affect firms’ profits, Saha and Goyal [26]and Sana [27] designed joint rebate, wholesale price discount, and cost-sharing contracts and a production inventory model. Based on the above literature analysis, we found that the implementation of CSR may be related to different subjects, and there are some differences in the implementation effect. In addition, the form of a supply chain contract may also affect the implementation of CSR.

Research on CM first appeared in the empirical marketing field, and the initial literature focused on the differences between CM and other traditional marketing methods. For example, Arora and Henderson [28] regarded CM products as a promotion strategy and compared CM with discounts, rebates, and other methods. In the case of low discounts, cause-linked products increase sales more effectively than discounts at the same price. Paul and Bloom [29] discussed how managers should allocate funds between CM and other types of marketing projects. In recent years, the empirical field has begun to focus on the impact of CM donations [30] and the purchase intentions of different consumers for different types of CM [31]. Chang [32] compared and analyzed two types of implementing CM through an empirical perspective and found that the donation ratio may be superior to the adoption of donation amount. Based on the inspiration from the field of empirical marketing regarding the different ways of implementing CM, we focus on the study of the optimization of CM implementation in the field of operation management by using donation ratio.

Research on CM in operation management began late. Krishna and Rajan [33] found that, when a firm implements CM, it increases the sales of cause-linked products and produces a spillover effect that drives the sales of other products. They constructed a simultaneous-action game model to discuss the optimal responses of different strategies. Heyes and Martin [34] combined firms’ products with social labels to study the impact of non-governmental organizations’ social labels on firms’ profits. Gao [5] developed a game-theoretic model to examine the pricing, design, and distribution of cause-linked products from the oligopoly perspective. Mallucci et al. [35] discuss the pricing decisions of cause-linked products under the influence of the dual effects of reputation and warm glow effects. Xu and Li [36] studied online retailers’ motivation to share demand information with suppliers that may implement CM strategies by comparing agency selling and reselling modes. Most aforementioned researches on the operation and management of CM are conducted from the perspective of donating part of the sales revenue or quantity to nonprofit organizations (NPOs). However, they do not describe the change in consumer utility caused by the implementation of CM from the perspective of donating a specific ratio of cause-linked products.

The above literature focuses on the pricing and donation decisions of CM under different scenarios. Unlike firms in the supply chain that use donation amounts to implement CM [5, 35, 36], firms that use donation ratio to implement CM are affected by the donation cost in our study, which in turn affects pricing. While previous studies [5, 33] on CM have been biased toward pricing, we divide the CM campaign mix into four modes according to different CM implementation subjects and the wholesale price (exogenous or endogenous), and we focus on optimizing and selecting different CM modes. Unlike some studies [24, 25] that focus only on CSR performance, the implementation of CM in our research focuses on the dual purpose of CSR and profit. In addition, by comparing the different donation and pricing results of CM modes, our study designs a donation cost-sharing contract that no previous CM research has mentioned.

The research most relevant to our study is that of Arya and Mittendorf [37], who mainly focus on donation and pricing decisions in government funding. In this study, we link the donation ratio to the warm glow effect generated by consumers. We analyze the CM pricing and donation decision results under four modes by constructing a supplier-led two-level supply chain Stackelberg game model. Through comparative analysis, we conclude who should implement CM in different cases and design a donation cost-sharing contract between the supplier and retailer. Thus, our results differ from Arya and Mittendorf.

## 3. Model description and the benchmark

### 3.1 Model description

Consider a supply chain comprising one supplier and one retailer. We assume that consumers are homogeneous and the unit production cost is *c*. The firm promises that, for each *q* quantity of cause-linked products sold, it will donate *βq* quantity (0<*β* ≤ 1) additional cause-linked products to NPOs. *β* is the donation ratio, through the analysis of CM cases using donation ratio, we found that it is relatively rare for firms to implement CM in a large donation ratio. The case where the donation ratio is larger and successful is the TOMS shoes selling one donate one CM campaigns [9]. In most cases, the donation ratio of firms to implement CM is not large (e.g., “Hodo”, “Patagonia”) [8, 32]. For these reasons, in our study, we assume that 0<*β* ≤ 1. The cause-linked products donated by the firm may be clothes, food, and so on [37]. Consistent with Gao [5], firms’ CM campaigns can be divided into different modes according to the implementation subjects and CM wholesale prices:

Mode 1 (M1): The wholesale price is exogenous. The supplier implements the CM and determines the donation ratio, and the retailer determines the retail price.
Mode 2 (M2): The wholesale price is exogenous. The retailer implements the CM and determines its donation ratio and retail price.
Mode 3 (M3): The wholesale price is endogenous. The supplier implements the CM and determines the donation ratio and wholesale price, and the retailer determines the retail price.
Mode 4 (M4): The wholesale price is endogenous. The retailer implements the CM, the supplier determines the wholesale price, and the retailer determines the donation ratio and retail price.

The notations of the parameters and variables used in this study are listed in Table 1.

**Table 1:** Notations of parameters and variables.

Decision variables	Descriptions
*p*	Retail price
*w*	Supplier’s wholesale price
*β*	Donation ratio; the firm will donate *βq* quantity for each *q* quantity of cause-linked products sold
**Parameters**	
*v*	Consumer’s valuation of product
*c*	Supplier’s unit production cost
*q*	Sales quantity
*μ*	The degree of preference for CM
*α*	The ratio of donation costs borne by the supplier
*Π*^*S*^, *Π*^*R*^	The profit of the supplier, retailer

#### 3.1.1 Warm glow effect function

When firms implement CM, consumers obtain additional utility through the ratio of product sales donated by firms, which is called the warm glow effect [2]. Koschate-Fischer et al. [30] proved empirically that the relationship between consumers’ willingness to pay and donation quantity is an increasing and concave function, that is, the warm glow effect function satisfies *γ*′(*β*) ≥ 0 and *γ*″(*β*) ≤ 0. Referring to Amaldoss et al. [38], we set the warm glow effect function as follows:

$$\gamma \left(\beta \right)=\mu \left(\beta -\frac{1}{2}\beta^{2}\right)$$ (1)

In (1), *β* = 0 corresponds to the case when there is no donation to the cause, and therefore *γ*(0) = 0. In addition, *lim*_*β*→1_ *γ*′(*β*) = 0 means that, when the donation ratio is large, the marginal utility of the warm glow effect decreases. Furthermore, *μ* (*μ* ≥ 0) is the degree of preference for CM [35], indicating the matching degree and importance of the CM campaign adopted by cause-linked products to the development of cause undertakings.

#### 3.1.2 Consumer utility function

As in Che [39] and Xiao and Shi [40], we assume that consumers’ perceived value of products is *v* ~ *U*[0,1]. When a firm implements CM, the consumer utility function is *u* = *v* − *p* + *γ*(*β*), *p* is the retail price of the cause-linked product, and *γ*(*β*) is the value of the warm glow effect caused by the firm donating *β* ratio cause-linked products’ market sales quantity *q*. The overall donation quantity of the firm is *d* = *βq*, and a consumer buys the product only when the consumer can achieve non-negative utility. The corresponding market demand is in *q* = 1 − *p* + *γ*(*β*).

### 3.2 The benchmark

We consider non-CM as the benchmark and compare the changes in the relevant parameters before and after the implementation of CM in the supply chain. Consider a supply chain composed of a single supplier and retailer without CM. The supplier decides the wholesale price *w* first, and then the retailer decides the retail price *p*. Finally, consumers buy products when their utility is non-negative.

The retailer’s profit function is

$$\Pi^{R}\left(p\right)=\left(p-w\right)\left(1-p\right)$$ (2)

The supplier’s profit function is

$$\Pi^{R}\left(w\right)=\left(w-c\right)\left(1-p\right)$$ (3)

Using the backward induction method in (2) and (3), we get the optimal wholesale price, retail price, and sales quantity are as follows:

$$w^{*}=\frac{\left(1+c\right)}{2},p^{*}=\frac{\left(3+c\right)}{4},q^{*}=\frac{\left(1-c\right)}{4}$$ (4)

The optimal profits of the retailer and supplier are as follows:

$$\Pi^{R*}=\frac{{\left(1-c\right)}^{2}}{16},\;\Pi^{S*}=\frac{{\left(1-c\right)}^{2}}{8}$$ (5)

## 4. Wholesale price exogenous

In this section, the parameter subscript “*ns*” indicates M1 and “*nr*” indicates M2.

### 4.1 The supplier implements CM (M1)

Our study discusses the donation and pricing decisions of supply chain CM from the two cases of endogenous or exogenous wholesale prices [5]. In M1, we consider the wholesale price contract to be usually long-term. Firms generally do not change their wholesale price when implementing CM in a short-term marketing campaign (e.g., Hodo). Thus, the wholesale price *w*_*ns*_ equals w* in (4). The decision sequence of the supplier implementing the CM under exogenous wholesale prices is as follows. First, the supplier’s wholesale price *w*_*ns*_ = w*, and the supplier sets the donation ratio *β*_*ns*_. Then, the retailer sets the retail price *p*_*ns*_, and, finally, when the consumers’ utility is non-negative, they will buy the cause-linked products.

The profit function of the retailer is

$$\Pi_{ns}^{R}\left(p_{ns}\right)=\left(p_{ns}-w^{*}\right)\left(1-p_{ns}+\mu \beta_{ns}-\frac{1}{2}\mu \beta_{ns}^{2}\right)$$ (6)

The profit function of the supplier is

$$\Pi_{ns}^{S}\left(\beta_{ns}\right)=\left(w^{*}-c\left(1+\beta_{ns}\right)\right)\left(1-p_{ns}+\mu \beta_{ns}-\frac{1}{2}\mu \beta_{ns}^{2}\right)$$ (7)

In Eq (7), the supplier’s profit equals the sales revenue minus the production cost of the total quantity of products produced by the supplier for sale and donation. Using backward induction to solve the optimization problem in (6) and (7), we have proposition 1.

**Proposition 1**. When the wholesale price is exogenous:

1. Only when *μ* > *c*, the supplier implements CM.
2. The optimal donation ratio and retail price are $\beta_{ns}^{*}=\frac{\mu +3c\mu -K}{6c\mu }$ and $p_{ns}^{*}=\frac{-\mu +K+3c\left(2\mu +c\left(16+8c+\mu \right)-K\right)}{72c^{2}}$, where $K=\sqrt{\mu \left(\mu +3c\left(-2\mu +c\left(4-4c+7\mu \right)\right)\right)}$.

Proposition 1 indicates that, in M1, the supplier implements CM only if the degree of preference for CM satisfies *μ* > *c*. Although the donation increases the supplier’s cost, it also causes a warm glow effect that can increase consumers’ utility and eventually increase its sales quantity. Therefore, the supplier implements CM only when the supplier’s profit from the increase in sales quantity compensates for the donation cost with a large enough preference for CM. The degree of preference for CM plays an essential role in its implementation; therefore, firms should pay attention to the method of measuring the degree of preference for CM. This can be inferred from the research of Krishna and Rajan [33].

**Property 1**. In M1, $p_{ns}^{*}>p^{*}$, $\;q_{ns}^{*}>q^{*}$, $\Pi_{ns}^{R*}>\Pi^{R*}\;$, and $\Pi_{ns}^{S*}>\Pi^{S*}$.

Property 1 shows that, in M1, the warm glow effect increases consumers’ utility and product price. The decrease in consumer utility caused by the price increase is less than the increase in consumer utility. Hence, the supplier’s CM implementation increases the product’s sales quantity and improves the retailer’s profits because the wholesale price is exogenous. When the degree of preference for CM is sufficiently large, the increase in revenue can compensate for the donation cost of the supplier, and it also increases the profit of the supplier in M1. Property 1 indicates that the retail price and sales quantity are larger than those without CM, which is also consistent with empirical market results, such as Koschate-Fischer et al. [30].

### 4.2 The retailer implements CM (M2)

In M2, the decision sequence of the retailer implementing CM under exogenous wholesale prices is as follows. First, the supplier adopts the wholesale price $w_{nr}^{*}=w^{*}$. Then, the retailer sets the retail price *p*_*nr*_ and the donation ratio *β*_*nr*_. Finally, when the consumers’ utility is non-negative, they will buy the cause-linked products.

The profit function of the retailer is

$$\Pi_{nr}^{R}\left(p_{nr},\beta_{nr}\right)=\left(p_{nr}-w^{*}\left(1+\beta_{nr}\right)\right)\left(1-p_{nr}+\mu \beta_{nr}-\frac{1}{2}\mu \beta_{nr}^{2}\right)$$ (8)

The retailer’s profit in Eq (8) is equal to the retailer’s sales revenue minus the retailer’s wholesale cost for the total quantity of products purchased from suppliers for sale and donation. From the retailer’s profit function, we obtain the optimal price $p_{nr}^{*}$ and the optimal donation ratio $\beta_{nr}^{*}$, so the supplier’s profit function is

$$\Pi_{nr}^{s}=(w^{*}-c)(1+\beta_{nr}^{*})(1-p_{nr}^{*}+\mu \beta_{nr}^{*}-\frac{1}{2}\mu \beta_{nr}^{*2})$$ (9)

By solving the optimization problem in (8) and (9), we have proposition 2.

**Proposition 2**. When the wholesale price is exogenous:

1. Only when *μ* > (1 + *c*)/2, the retailer implements CM.
2. The optimal donation ratio and the optimal retail price are $\beta_{nr}^{*}=1-\frac{1+c}{2\mu },\;p_{nr}^{*}=\frac{1}{16}(8(2+c)-\frac{3(1+c{)}^{2}}{\mu }+4\mu )$.

Similar to proposition 1, in M2, the retailer implements CM only if its degree of preference for CM satisfies *μ* > (1 + *c*)/2. If the revenue increase caused by the warm glow effect compensates for the donation cost, the retailer implements CM. However, in contrast to proposition 1, the retailer’s unit donation cost is the wholesale price (1 + *c*)/2, and the supplier’s unit donation cost is the unit production cost *c*, which means that it is easier for the supplier to implement CM than the retailer.

**Property 2**. In M2, $p_{nr}^{*}>p^{*},\;q_{nr}^{*}>q^{*},\;\Pi_{nr}^{R*}>\Pi^{R*}$, and $\Pi_{nr}^{S*}>\Pi^{S*}$.

Property 2 shows that, in the case of M2, the warm glow effect also increases retail price and sales quantity. Due to the exogenous wholesale price, the increase in sales quantity will lead retailers to order more products from suppliers, thereby increasing the suppliers’ profit. When the degree of preference for CM is sufficiently large, the increase in revenue compensates for the donation cost of the retailer, and the profits of the retailer also increase.

### 4.3 Comparative analysis

Comparing the analysis of M1 and M2, the supplier, as the dominant supply chain player, should consider whether the implementation of CM by itself or the retailer is more beneficial for improving profits. The relevant propositions and properties are as follows.

**Property 3**. (1) $\beta_{ns}^{*}$ and $\beta_{nr}^{*}$ are increasing functions of *μ* and decreasing functions of *c*, respectively.

(2) When $c<\mu \leq \frac{1-c(4+c)+\sqrt{1+c(c(26+c(32+13c))-8)}}{4c},\;\beta_{ns}^{*}\geq \beta_{nr}^{*}$; when $\mu >\frac{1-c(4+c)+\sqrt{1+c(c(26+c(32+13c))-8)}}{4c},\;\beta_{ns}^{*}\leq \beta_{nr}^{*}$.

It is easier to prove $\partial \beta_{ns}^{*}/\partial \mu >0,\;\partial \beta_{ns}^{*}/\partial c<0,\;\partial \beta_{nr}^{*}/\partial \mu >0$, and $\partial \beta_{nr}^{*}/\partial c<0$. Assuming $\beta_{ns}^{*}=\beta_{nr}^{*}$, we obtain property 3(2). Property 3(1) indicates that the optimal donation ratio increases with an increase in the degree of preference for CM and decreases with an increase in the unit production cost. This is because, when the degree of preference for CM is larger, the supplier or retailer will set a larger donation ratio to earn a warmer glow effect. Thus, the sales quantity and retail price will increase, and the profit of the supplier or retailer will improve. However, the warm glow effect generated by increasing the donation ratio increases the profit of the supplier or retailer, which cannot compensate for the decrease in the profit of the supplier or retailer caused by the increase in donation cost. Therefore, the supplier or retailer should reduce the donation ratio to ensure profits.

Property 3(2) indicates that, when the degree of preference for CM *μ* is within a specific range, the donation ratio of the supplier implementing the CM is larger than that of the retailer implementing the CM. In addition, while the preference degree for CM is large, the donation ratio of the supplier implementing CM is smaller than that of the retailer implementing CM. This is because the lower donation cost causes the supplier to implement CM at a lower CM preference degree. The donation will increase sales quantity, improving the supplier’s profits even if the wholesale price does not change. However, when the degree of preference for the CM is higher, the supply chain power shifts to the retailer. The retailer sets a larger donation ratio to obtain a warmer glow effect. An increase in the warm glow effect leads to an increase in sales quantity, thereby increasing the profits of both parties. When the degree of preference for CM is sufficiently large, the donation ratio of M2 is greater than that of M1. However, with the increase in unit production cost, the range of the larger donation ratio of the supplier narrows; that is, the possibility of implementing CM by the retailer with a larger unit production cost increases.

**Proposition 3**. When the wholesale price is exogenous:

1. When *μ* ∈ [0, *c*], neither the supplier nor the retailer implements the CM.
2. When *μ* ∈ [0, *μ*_1_] and $\Pi_{ns}^{S*}\geq \Pi_{nr}^{S*}$, the supplier should implement the CM.
3. When *μ* ∈ [*μ*_1_, *μ*_2_], $\Pi_{ns}^{S*}<\Pi_{nr}^{S*}$, and $\Pi_{ns}^{R*}<\Pi_{nr}^{R*}$, the supplier should implement CM.
4. When *μ* > *μ*_2_, $\Pi_{ns}^{S*}<\Pi_{nr}^{S*}$, and $\Pi_{ns}^{R*}<\Pi_{nr}^{R*}$, the retailer should implement CM.

*μ*_1_ satisfies $27c^{2}(-1+c)(1+c-4\mu_{1})((1+c{)}^{2}-8c\mu_{1}+4\mu_{1}^{2})-4\mu_{1}((2-6c)\mu_{1}^{2}+K)(\mu -K+3c(4c^{2}-2\mu_{1}^{2}-c(4+\mu_{1}^{2})+K))=0$, and *μ*_2_ satisfies $72c^{2}((1+c{)}^{2}-8c\mu_{2}+4\mu_{2}^{2})-16\mu_{2}(K-\mu_{2}-3c(4c^{2}-2\mu_{2}-c(4+\mu_{2})+K))=0$.

Assuming $\Pi_{ns}^{S*}=\Pi_{nr}^{S*}$ and $\Pi_{ns}^{R*}=\Pi_{nr}^{R*}$, we obtain the degree of preference for CM *μ*_1_ and *μ*_2_, respectively. Proposition 3(1) shows that, when the degree of preference for CM is small, neither the supplier nor the retailer’s increased profits from implementing CM compensate for the donation cost. Proposition 3(2) shows that, when *μ* ∈ [*c*, *μ*_1_], the supplier makes more profit by implementing CM than when the retailer implements CM or neither. Therefore, the supplier should take the initiative to implement CM. Proposition 3(3) shows that, when *μ* ∈ [*μ*_1_, *μ*_2_], we obtain $\Pi_{ns}^{S*}<\Pi_{nr}^{S*}$ and $\Pi_{ns}^{R*}>\Pi_{nr}^{R*}$, and either the supplier or retailer wants the other to implement CM to make it more profitable. As the leader in the supply chain, the supplier should take the initiative to implement CM, and the retailers use free-riding to make more profit. However, the supplier shares the donation cost with the retailer to curb the free-riding behavior of the retailer in the implementation of CM to achieve a win-win situation. This is because the implementation of CM by the retailer is equivalent to optimizing the ratio of donations and retail prices from the perspective of a centralized supply chain. When the degree of preference for CM is relatively large, the retailer implementing the CM will increase both the retailer’s and the supplier’s profits. Proposition 3(4) shows that, when $\mu >\mu_{2},\;\Pi_{ns}^{S*}<\Pi_{nr}^{S*}$, and $\Pi_{ns}^{R*}<\Pi_{nr}^{R*}$ occur, the implementation of CM by the retailer is beneficial for both the retailer and supplier.

**Fig 1:**
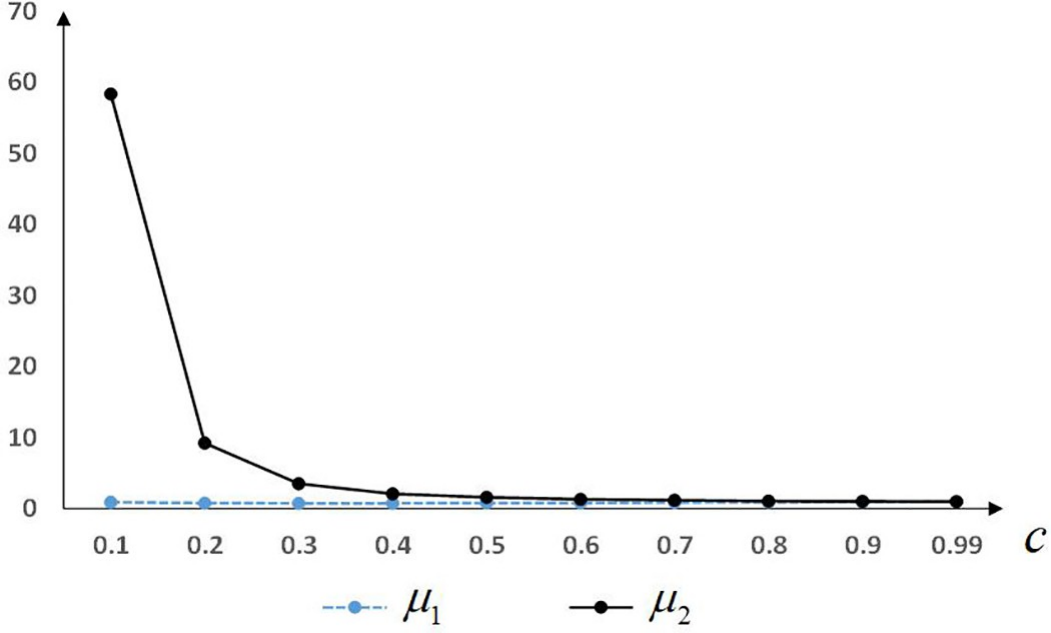
Relationship between *μ*_1_, *μ*_2_ and the unit production cost *c*.

As the equilibrium solutions of *μ*_1_ and *μ*_2_ are implicit functions, the relationship between the magnitude of *μ*_1_, *μ*_2_, and unit production cost *c* is shown in Fig 1.

Fig 1 shows that, when 0<*c* < 1, we get *μ* > *μ*_2_. Fig 1 also shows that, when the unit production cost *c* is low, the retailer’s implementation of CM requires a high degree of preference for CM *μ*. That is, cause-linked products with lower unit production costs are not suitable for retailers to implement CM. When the unit production cost *c* is low, the gap between the donation cost (1 + *c*)/2 in M2 and *c* in M1 increases. The retailer’s advantage in implementing CM is evident when the degree of preference for the CM is sufficiently large. Moreover, for cause-linked products with higher unit production costs, the gap between the donation cost of suppliers or retailers to implement CM is not large, and the retailer’s probability of implementing CM increases.

**Fig 2:**
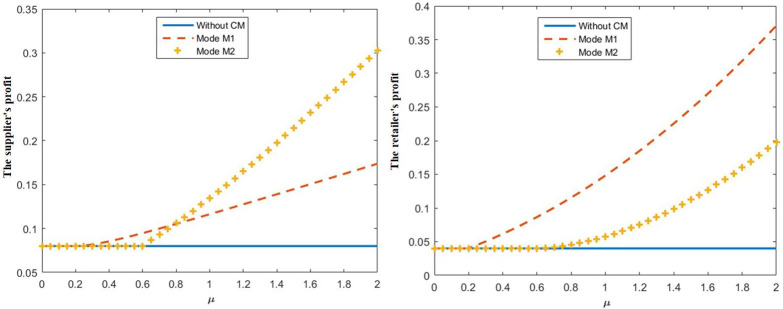
The influence of *μ* on the supplier’s profit and retailer’s profit.

When *c* = 0.2, taking *μ* ∈ [0,2], we obtain the relationship between the degree of preference for CM *μ* and the optimal supplier’s and retailer’s profit, as shown in Fig 2. We find that, only when *μ* > 0.2, the supplier implements CM; only when *μ* > 0.6, the retailer implements CM; and the profits of the supplier and retailer in M1 and M2 are larger than in the case without CM, consistent with properties 1 and 2. We also find that, during *μ* ∈ [0.2,0.79], the supplier should implement CM. When *μ* > 0.79 is implemented, the supplier profit of the retailer implementing CM is larger than that of the supplier implementing CM. However, at this time, the retailer will not implement CM; if the supplier can use a donation cost-sharing contract to make the retailer implement CM, it may achieve a win-win situation.

### 4.4 Donation cost-sharing contract

We design a donation cost-sharing contract that guarantees that the retailer’s profit in implementing CM is not lower than the profit of the retailer in the implementation of CM by the supplier to avoid the occurrence of free-riding behavior of retailers when the wholesale price is exogenous. “*jr*” indicates that the retailer implements CM, and the supplier and retailer jointly share the donation costs when the wholesale price is exogenous (M5). The difference between the decision sequences of M5 and M2 is that the supplier determines the donation cost ratio *α*(0 < *α* < 1).

The profit function of the retailer is

$$\Pi_{jr}^{R}\left(p_{jr},\beta_{jr}\right)=\left(p_{jr}-w_{jr}\right)\left(1-p_{jr}+\gamma \left(\beta_{jr}\right)\right)-\left(1-\alpha \right)\beta_{jr}w_{jr}\left(1-p_{jr}+\gamma \left(\beta_{jr}\right)\right)$$ (10)

According to the optimization results of the retailer’s profit function, we obtain the optimal retail price $p_{jr}^{*}$ and the optimal donation ratio $\beta_{jr}^{*}$, and the supplier’s profit function is

$$\Pi_{jr}^{S}\left(\alpha \right)=\left(w^{*}-c\right)\left(1-p_{jr}^{*}+\gamma \left(\beta_{jr}^{*}\right)\right)\left(1+\beta_{jr}^{*}\left(1-\alpha \right)\right)-c\alpha \beta_{jr}^{*}\left(1-p_{jr}^{*}+\gamma \left(\beta_{jr}^{*}\right)\right)$$ (11)

In (10) and (11), the donation cost ratio must meet $\Pi_{jr}^{S}\left(\alpha \right)>max\{\Pi_{ns}^{S*},\Pi^{S*}\}$ and $\Pi_{jr}^{R}\left(\alpha \right)>\Pi_{ns}^{R*}$. The first condition ensures that the supplier’s profit in M5 is greater than in M1 and the base model. The second condition ensures that the retailer’s profit in M5 is greater than that in M1.

**Proposition 4**. When the wholesale price is exogenous and *μ* ∈ [*μ*_1_, *μ*_2_]:

1. Only when *μ* > (1 − *α*)(1 + *c*)/2 and *α* ∈ [*α*_3_, min⁡{*α*_1_, *α*_2_}], the retailer implements CM with the condition of a donation cost-sharing contract.
2. The optimal donation ratio and the optimal retail price are $\beta_{jr}^{*}=1-\frac{\left(1-\alpha )(1+c\right)}{2\mu }$ and $p_{jr}^{*}=\frac{4\mu^{2}-3{\left(1-\alpha \right)}^{2}{\left(1+c\right)}^{2}-4\mu (-4+\alpha -2c+\alpha c)}{16\mu }$.

*α*_1_, *α*_2_, *α*_3_ are the results obtained by making $\Pi_{jr}^{S}\left(\alpha_{1}\right)=\Pi_{ns}^{S*},\;\Pi_{jr}^{S}\left(\alpha_{2}\right)=\Pi^{S*}$, and $\Pi_{jr}^{R}\left(\alpha_{3}\right)=\Pi_{ns}^{R*}$.

Proposition 4 reveals that, in M5, the retailer’s implementation of CM requires a lower degree of preference for CM than in M2. When the supplier shares the cost of donations, the retailer implements CM with a lower degree of preference for CM because of the lower donation cost. Proposition 4 also reveals that the donation cost ratio needs to be within a specific range for the retailer to have an incentive to implement CM. As the donation cost ratio increases, the retailer’s donation cost of implementing CM decreases. Therefore, a larger ratio of cause-linked products will be donated to capture the warm glow effect, increasing sales quantity and retail price, thereby improving the retailer’s profit. However, for the supplier, when the ratio of donations borne by the supplier brings less profit than when the supplier implements CM by itself, the supplier will not choose to undertake the donation contract jointly. Therefore, the ratio of donation costs borne by suppliers need to be within a specific range.

**Fig 3:**
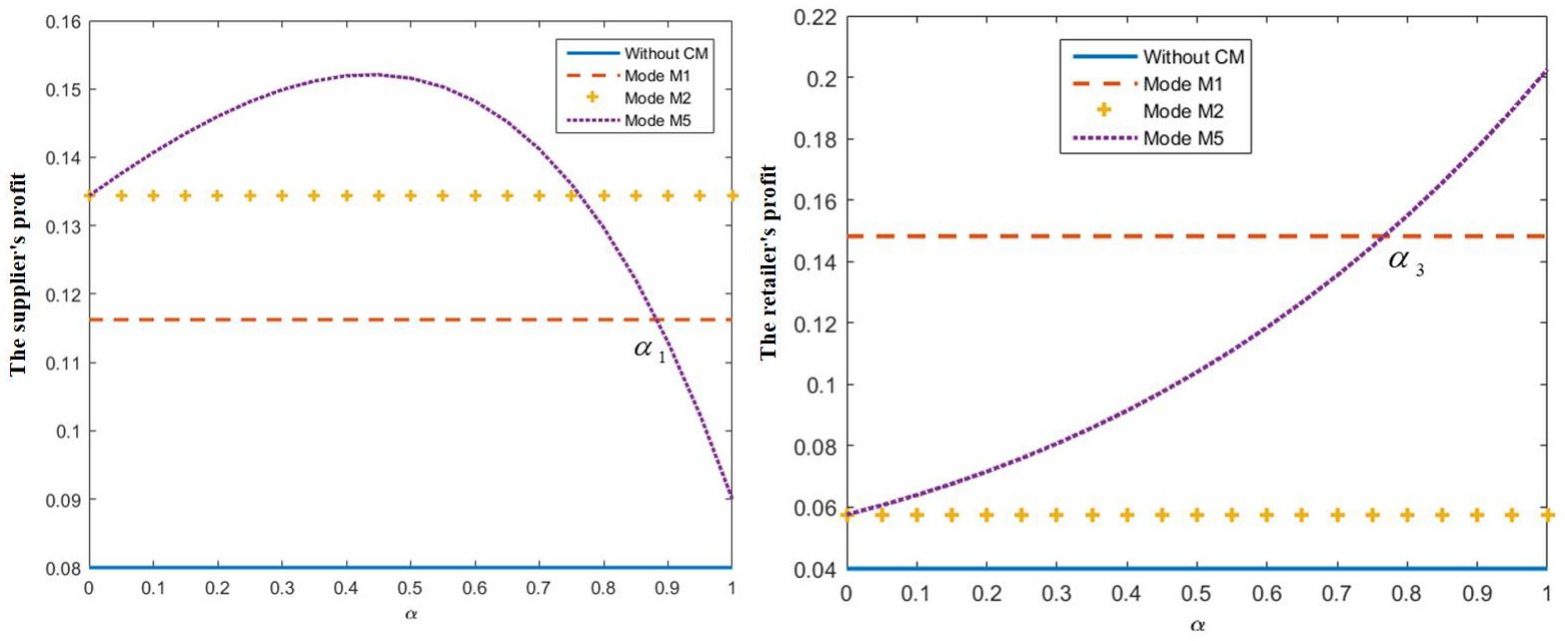
The influence of *α* on the supplier’s and retailer’s profit.

When *c* = 0.2 and *μ* = 1, taking *α* ∈ [0,1], we obtain the relationship between the donation cost ratio *α* and the optimal supplier’s profit and retailer’s profit, as shown in Fig 3. We find that, when *μ* = 1, we obtain $\Pi_{ns}^{S*}<\Pi_{nr}^{S*}$ and $\Pi_{ns}^{R*}<\Pi_{nr}^{R*}$, and, when *α* < *α*_1_ = 0.88, the supplier’s profit is greater in M5 than in M1. However, suppose that the retailer’s profit is smaller in M5 than in M1; in this case, the retailer will not implement the CM. Therefore, *α* > *α*_3_ = 0.77 must be satisfied before the retailer implements CM. In the numerical analysis, we obtained *α*_2_ > 1, so it is only necessary for *α* ∈ [0.77,0.88]. The supplier and the retailer sharing the donation cost will make the retailer’s and the supplier’s profits greater than the supplier’s implementation of CM. These results are consistent with proposition 4.

In summary, in the case of exogenous wholesale prices, the supplier decides to implement CM based on the degree of preference for CM. In addition, to avoid the free-riding behavior of the retailer in the implementation of CM, the supplier, as the leader in the supply chain, should actively bear donation costs to encourage the retailer to implement CM.

## 5. Wholesale prices endogenous

In this section, the parameter subscript “*cs*” indicates M3, and “*cr*” indicates M4.

### 5.1 The supplier implements CM (M3)

When the wholesale price is endogenous and the supplier implements CM, the decision sequence differs from that of M1 in that the supplier determines the wholesale price *w*_*cs*_.

The profit function of the retailer is

$$\Pi_{cs}^{R}\left(p_{cs}\right)=\left(p_{cs}-w_{cs}\right)\left(1-p_{cs}+\mu \beta_{cs}-\frac{1}{2}\mu \beta_{cs}^{2}\right)$$ (12)

The profit function of the supplier is

$$\Pi_{cs}^{S}\left(w_{cs},\beta_{cs}\right)=\left(w_{cs}-c\left(1+\beta_{cs}\right)\right)\left(1-p_{cs}+\mu \beta_{cs}-\frac{1}{2}\mu \beta_{cs}^{2}\right)$$ (13)

Using backward induction to solve the optimization problem in (12) and (13), we have proposition 5.

**Proposition 5**. When the wholesale price is endogenous:

1. Only when *μ* > *c*, the supplier implements CM.
2. The optimal donation ratio, retail price, and wholesale price are $\beta_{cs}^{*}=1-\frac{c}{\mu },\;p_{cs}^{*}=\frac{1}{8}(6+4c-\frac{{5c}^{2}}{\mu }+3\mu )$, $w_{cs}^{*}=\frac{1}{4}(2+4c-\frac{{3c}^{2}}{\mu }+\mu )$.

Similar to Proposition 1, as the supplier’s donation cost in implementing CM does not change, proposition 5 shows that, regardless of whether the wholesale price is endogenous or exogenous, the supplier implements CM only when the increase in revenue due to the warm glow effect compensates for the supplier’s donation cost. However, the difference is that the revenue generated by the warm glow effect when the wholesale price is endogenous is due to an increase in sales quantity and wholesale price.

**Property 4**. In M3, $p_{cr}^{*}>p^{*},\;w_{cs}^{*}>w^{*},\;q_{cs}^{*}>q^{*},\;\Pi_{cs}^{R*}>\Pi^{R*}$, and $\Pi_{cs}^{S*}>\Pi^{S*}$.

Property 4 shows that, in M3, the optimal retail price, wholesale price, sales quantity, and profit for suppliers and retailers are all larger than those without implementing CM. Thus, in M3, the supply chain has a double marginal effect; the supplier and retailer only consider their marginal benefits when making decisions. As the donation increases the marginal cost of the product, the supplier needs to increase the wholesale price to obtain maximum profit; the increase in the wholesale price also increases the retailer’s marginal cost. Therefore, the retailer needs to increase the retail price to obtain maximum profit, leading to higher wholesale and retail prices than when CM is not implemented. However, the warm glow effect caused by donations increases product utility and product sales, compensating for the increased cost of donations, thereby increasing the profits of the supplier and retailer.

### 5.2 The retailer implements CM (M4)

When the wholesale price is endogenous and the retailer implements CM, the decision sequence differs from M2, because the supplier determines the wholesale price *w*_*cr*_.

The profit function of the retailer is

$$\Pi_{cr}^{R}\left(p_{cr},\beta_{cr}\right)=\left(p_{cr}-w_{cr}\left(1+\beta_{cr}\right)\right)\left(1-p_{cr}+\mu \beta_{cr}-\frac{1}{2}\mu \beta_{cr}^{2}\right)$$ (14)

The profit function of the supplier is

$$\Pi_{cr}^{S}\left(w_{cr}\right)=\left(w_{cr}-c\right)\left(1+\beta_{cr}\right)\left(1-p_{cr}+\mu \beta_{cr}-\frac{1}{2}\mu \beta_{cr}^{2}\right)$$ (15)

By solving the optimization problem in (14) and (15), we have proposition 6.

**Proposition 6**. When the wholesale price is endogenous:

1. Only when $\mu >w_{cr}^{*}$, the retailer implements CM.
2. The optimal donation ratio and the optimal retail price are $\;\beta_{cr}^{*}=1-\frac{w_{cr}^{*}}{\mu },\;p_{cr}^{*}=\frac{1}{4}(2+\mu -\frac{3w_{cr}^{*}}{\mu }+{4w}_{cr}^{*})$.

$w_{cr}^{*}$ satisfies $\mu (4\mu +2\mu^{2}+2c+9c\mu )-2\mu \left(2+6c+9\mu \right)w_{cr}^{*}+3\left(c+6\mu \right)w_{cr}^{*2}-4w_{cr}^{*3}=0$.

Proposition 6 shows that, in M4, when the retailer implements CM, the warm glow effect also increases consumer utility. However, unlike M3, we cannot obtain the results of $p_{cr}^{*}>p^{*}$. The main reason for this is that, when the retailer implements CM, the increase in the retailer’s profit depends on the price and sales quantity. The cost of the retailer’s donations depends on the supplier’s wholesale price. The retailer then has two ways to increase profits. The first is to reduce retail prices and increase profits by increasing sales quantity. This further prompts the supplier to lower wholesale prices and indirectly reduce the retailer’s donation costs. The second method is to increase the retail price to improve profits. When the retailer implements CM, it chooses a method to increase profits, mainly depending on the size of the warm glow effect. When the degree of preference for the CM is small, increasing the retail price will not improve the utility of the product, but some consumers are lost. Then, the retailer chooses to reduce the price to increase the sales quantity, prompting the supplier to reduce the wholesale price to share the donation cost, thereby indirectly increasing profit. When the degree of preference for the CM is large, the product’s utility is greatly improved, and it is appropriate to increase the retail price of the product to promote the retailer’s profit.

### 5.3 Comparative analysis

By comparing and analyzing the different modes of CM donation decisions, we obtain property 5.

**Property 5**. (1) $\beta_{cs}^{*}\;$ is an increasing function of *μ* and decreasing functions of *c*.

(2) $\beta_{cs}^{*}>max\{\beta_{cr}^{*},\beta_{ns}^{*},\beta_{nr}^{*}\}$.

Property 5 (1) is easy to prove, and the correlation analysis is the same as in property 3. From Property 5 (2), we obtain the $\beta_{cs}^{*}>max\{\beta_{cr}^{*},\beta_{ns}^{*},\beta_{nr}^{*}\}$. When the wholesale price is endogenous, the supplier is the leader in the supply chain, and the donation cost is lower. Hence, the supplier obtains a higher warm glow effect by donating a larger ratio of products to maximize profits. Therefore, the donation ratio is larger in M3 than in M4. For M1, the supplier increases the donation ratio to obtain the warm glow effect by increasing only the sales quantity and not the wholesale price. However, in M3, the supplier improves profits by increasing wholesale price and sales quantity. Therefore, the supplier donates a larger ratio in M3 than in M1 to obtain a warm glow effect. In M2, although the retailer implements CM from a centralized supply chain perspective, the supplier can rely only on the increase in sales quantity brought about by the warm glow effect to improve profits. Therefore, the donation ratio in M3 is larger than that in M2. A comparative analysis of the ratio of donations helps firms implement CM and guide NPOs to find the type of companies to cooperate with to obtain more donated materials.

**Fig 4:**
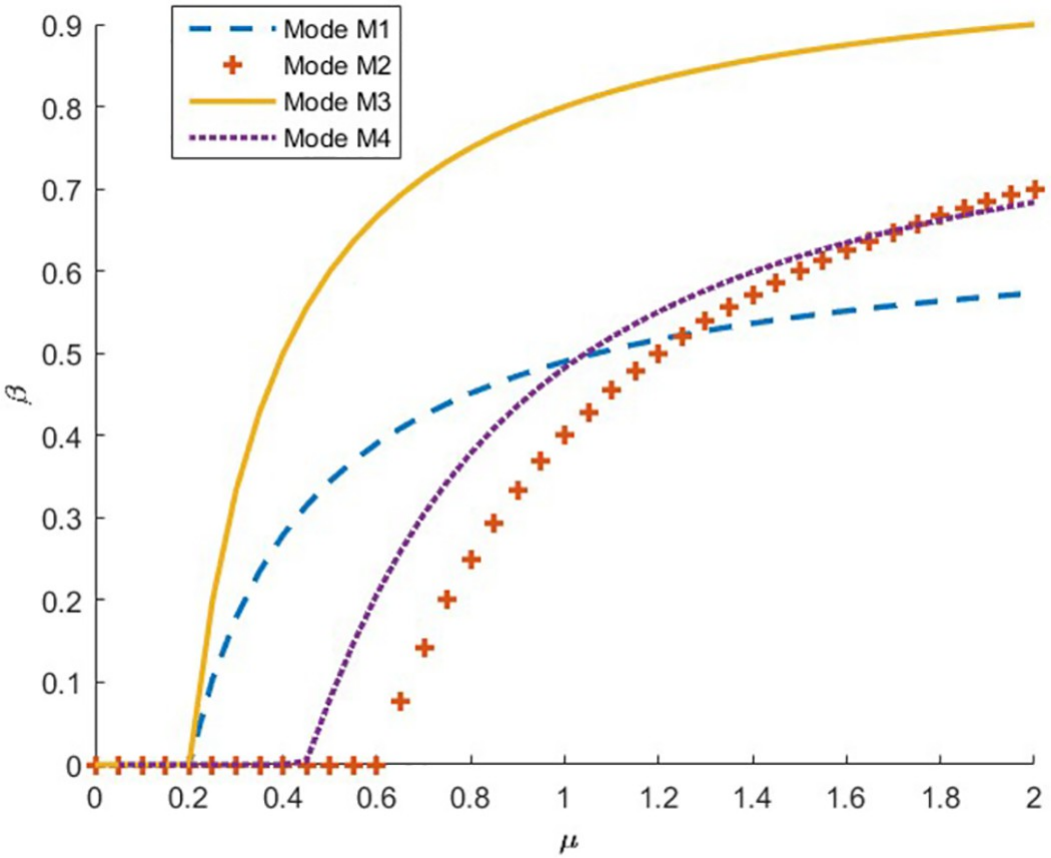
The influence of parameters *μ* on donation ratio.

When *c* = 0.2, taking *μ* ∈ [0,2], we obtain the relationship between the degree of preference for CM *μ* and optimal donation ratio, as shown in Fig 4. We find that, when *μ* ∈ [0.2,1.26], the donation ratio in M1 is larger than that in M2, and the opposite is true when *μ* ∈ [1.26,2]. The donation ratio in M3 is always larger than that in M4. Compared with the wholesale price exogenous case, the donation ratio in M3 is larger than that in M1 and M2, which is also consistent with property 5.

Proposition 3 provides the supplier’s optimal decision in different ranges of the CM preference degree under the exogenous wholesale price condition. Thus, for endogenous wholesale prices, the supplier should also consider whether to implement CM by itself or with the retailer to obtain greater profits. However, because the optimal wholesale price in M4 is an implicit function, it is impossible to compare the size of the analytical solution between the optimal supplier’s and retailer’s profits in M3 and M4. Next, we compare the optimal supplier’s and retailer’s profits in M3 and M4 through detailed qualitative and numerical simulation analyses.

When the wholesale price is endogenous, the retailer guarantees profit by changing the retail price, and the supplier guarantees profit by changing the wholesale price. Therefore, any party implementing the CM will ensure profit maximization. In addition, because the cost of implementing CM by the supplier is low, the supplier’s profit will be larger when the supplier implements CM than when the retailer implements CM. Therefore, when the wholesale price is endogenous, the supplier should take the initiative to implement the CM.

**Fig 5:**
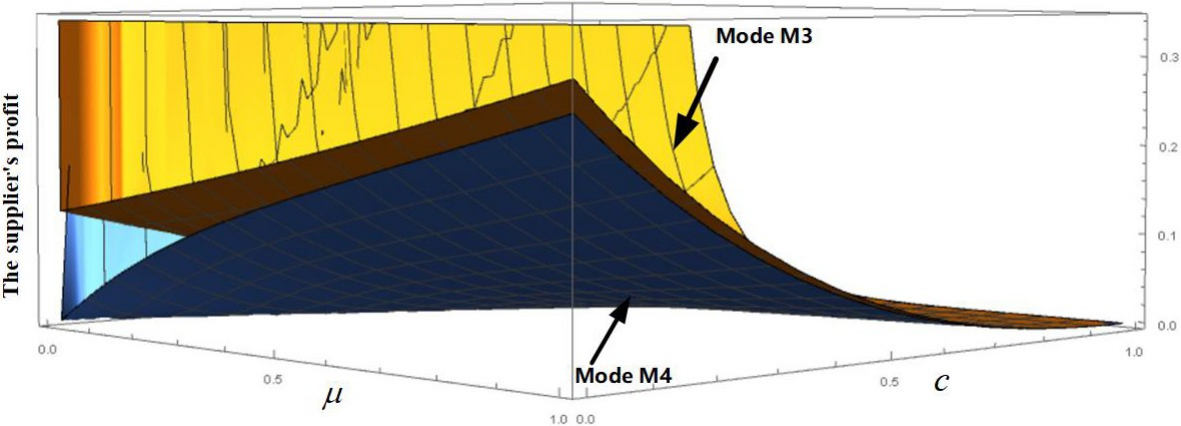
The supplier’s profit of supplier or retailer to implement CM with endogenous wholesale prices.

We use numerical analysis to verify the foregoing analysis results. Taking *c* ∈ [0,1] and *μ* ∈ [0,1], we obtain a surface plot of the supplier’s profit in M3 and M4, as shown in Fig 5. Fig 5 shows that, in the case of the endogenous wholesale price, the profit of the supplier implementing CM is always larger than that of the retailer implementing CM within the scope of the relevant parameters. Therefore, the supplier should take the initiative to implement CM to meet the preconditions for CM. For example, Chando actively implements CM, which is more beneficial to its interests.

The foregoing corresponding analysis results will inspire management when the wholesale price is endogenous. Suppliers should be more active in implementing CM. Firms should be encouraged to conduct CM and formulate relevant subsidy policies. NPOs should try to select companies that implement CM over a long period to obtain a larger ratio of donated materials. When consumers choose cause-linked products, they should pay more attention to the donation ratio of the firm than to the total revenue or quantity.

## 6. Discussion and conclusion

### 6.1 Discussion

As the CM campaign in the implementation of CSR strategies is becoming increasingly widespread, many firms have begun to pay attention to implementing CM campaigns, but how to optimize and select the CM mode is an urgent problem faced by firms. We divided the CM campaign into four modes based on the different implementation subjects of CM and endogenous or exogenous wholesale prices. We compared the donation and pricing decisions of different modes of CM in a two-tier supplier-led supply chain consisting of a single supplier and a single retailer. We explored the inherent characteristics of the CM mode by constructing a warm glow effect model based on the donation ratio. In addition, we constructed a supply chain CM Stackelberg game model to determine the optimal pricing and donation decisions for the implementation of CM to maximize profits.

Based on our analysis, we generated the results of the supply chain firms’ choice of the CM mode, as summarized in Table 2. Table 2 shows that, when the degree of preference for CM is sufficiently large, the income generated by the increase in sales quantity and retail price compensates for the donation cost, and the supplier or retailer can implement CM. In the case of exogenous wholesale prices, when the degree of preference for CM is relatively low, the supplier should implement CM, and when the degree of preference for CM is relatively high, the retailer should implement CM. When the degree of preference for CM is moderate, the supplier suppresses the free-rider behavior of the retailer in implementing CM by sharing donation costs with the retailer, thereby achieving a win-win situation. In the case of endogenous wholesale prices, the supplier should take the initiative to implement the CM.

**Table 2:** Summary of the main results.

CM decision	The supplier	The retailer
Case of wholesale price		
Exogenous	If *μ* ∈ [*c*, *μ*_1_], the supplier implements CM	If *μ* ∈ [*μ*_1_, *μ*_2_], the retailer implements CM with a donation cost-sharing contract
		If *μ* > *μ*_2_, the retailer implements CM
Endogenous	If *μ* > *c*, the supplier implements CM	It’s not suitable for the retailer to implement CM at any time

### 6.2 Conclusion

Through research, we found that whether a firm implements CM depends on the size of the warm glow effect. And firms can implement CM only when the benefits from increased sales and prices due to the warm glow effect can compensate for donation costs, which will lead to higher profits than if they do not implement CM. As the donation cost of retailers is larger than that of suppliers, it is easier for suppliers to implement CM. The donation ratio is an increasing function of the degree of preference for CM and a decreasing function of the unit production cost. And the donation ratio is the largest in the case of suppliers implementing CM at endogenous wholesale prices. In the case of the exogenous wholesale price, the supplier-led supply chain should preferentially select the implementation subjects (supplier or retailer) of CM according to the different ranges of the degree of preference for CM. To avoid free-riding behavior by retailers in implementing CM, suppliers should proactively share the cost of donations with retailers to achieve a win-win outcome. In the case of the endogenous wholesale price, suppliers should take the initiative to implement CM.

Our findings offer practitioners interesting managerial insight. From the firm’s perspective, if the wholesale price contract cannot be changed in a short period, when the degree of preference for CM is relatively low, the supplier should implement the CM. And when the degree of preference for the CM is within a specific range, suppliers, as the leading party of the supply chain, should actively bear the donation cost and encourage retailers to implement CM. However, if the wholesale price contract can be changed, suppliers should take the initiative to implement CM. From an NPO’s perspective, they should cooperate with firms that have been prepared to implement CM campaigns for a long time to obtain a larger ratio of donations. From the consumer’s perspective, when choosing cause-related products, consumers should pay more attention to the donation ratio of the unit sold rather than the unit donation amount, which is more conducive to accurately measuring the actual contribution of firms’ implementation of CM. From the government’s perspective, it should focus on the ratio of firm donations to formulate relevant subsidy policies rather than just the total amount.

Although some management insights have been obtained into the implementation of CM for firms, there are several possible directions for future research. First, we assume that all consumers are homogeneous when the firm implements CM. Nevertheless, if we consider the heterogeneity of consumers [5], how should a firm make donations and pricing decisions? In addition, government subsidies can prompt firms to implement CM campaigns, and our future research can extend the model to research CM decision-making under government subsidies. Finally, we focus on the donation and pricing decisions of the supplier-led two-level supply chain implementation of CM. However, with the rapid development of intelligent platforms such as JD.com and Tmall, other retail platform-led CM campaigns are becoming increasingly common. During the COVID-19 pandemic, major e-commerce platforms are driving rapid economic recovery in remote mountainous areas through the implementation of CM. Therefore, we will focus on research on CM decision-making led by intelligent platforms in the future.

## Supporting information

S1 Appendix (DOCX)

## References

[1] Andrews M, Luo X, Fang Z, Aspara J. Cause Marketing Effectiveness and the Moderating Role of Price Discounts. J Marketing. 2014;78(6):120–42.

[2] Andreoni J. Giving with Impure Altruism: Applications to Charity and Ricardian Equivalence. The Journal of political economy. 1989;97(6):1447–58.

[3] Varadarajan PR, Menon A. Cause-Related Marketing: A Coalignment of Marketing Strategy and Corporate Philanthropy. J Marketing. 1988;52(3):58–74.

[4] Yang L, Yang YZ. Characteristics and strategies of brand public service communication in the new media environment. New Media Research. 2020;6(12):117–9. (In Chinese)

[5] Gao F. Cause Marketing: Product Pricing, Design, and Distribution. Manufacturing & service operations management. 2020;22(4):775–91.

[6] Cachon GP. Supply Chain Coordination with Contracts.: Elsevier B.V; 2003. p. 227–339.

[7] Lariviere MA, Porteus EL. Selling to the Newsvendor: An Analysis of Price-Only Contracts. Manufacturing & service operations management. 2001;3(4):293–305.

[8] Hodo "selling goose down clothes in dog days" JD crowdfunding cause marketing. IAI AWARDS. 2018. https://www.iaiad.com/en/case_en/19th-iai-award/19th-silver/1594.html. (In Chinese)

[9] Wydick B, Katz E, Calvo F, Gutierrez F, Janet B. Shoeing the Children: The Impact of the TOMS Shoe Donation Program in Rural El Salvador. The World Bank economic review. 2018;32(3):727–51.

[10] Davis K. Social Responsibility Is Inevitable. Calif Manage Rev. 1976;19(1):14–20.

[11] Tang CS. Socially responsible supply chains in emerging markets: Some research opportunities. J Oper Manag. 2018;57(1):1–10.

[12] Sodhi MS, Tang CS. Social enterprises as supply-chain enablers for the poor. Socio-Econ Plan Sci. 2011;45(4):146–53.

[13] Berenguer G, Shen ZM. OM Forum—Challenges and Strategies in Managing Nonprofit Operations: An Operations Management Perspective. Manufacturing & service operations management. 2020;22(5):888–905.

[14] Besiou M, Van Wassenhove LN. Humanitarian Operations: A World of Opportunity for Relevant and Impactful Research. Manufacturing & service operations management. 2020;22(1):135–45.

[15] Sana SS. Price competition between green and non green products under corporate social responsible firm. J Retail Consum Serv. 2020;55:102118.

[16] Du S, Wang L, Hu L, Zhu Y. Platform-led green advertising: Promote the best or promote by performance. Transportation research. Part E, Logistics and transportation review. 2019;128:115–31.

[17] Xie J, Liu D, Liang L, Li Q. Contract choice and advance selling strategy in a supply chain of FAP. Plos One. 2022;17(3):e265661. 10.1371/journal.pone.0265661 PMC8947360 35324980

[18] Forghani E, Sheikh R, Hosseini SMH, Sana SS. The impact of digital marketing strategies on customer’s buying behavior in online shopping using the rough set theory. International journal of system assurance engineering and management. 2021;13(2):625–40.

[19] Nielsen IE, Majumder S, Sana SS, Saha S. Comparative analysis of government incentives and game structures on single and two-period green supply chain. J Clean Prod. 2019;235:1371–98.

[20] Pal B, Sana SS, Chaudhuri K. Two-echelon competitive integrated supply chain model with price and credit period dependent demand. Int J Syst Sci. 2016;47(5):995–1007.

[21] Xu X, Zhang M, Dou G, Yu Y. Coordination of a supply chain with an online platform considering green technology in the blockchain era. Int J Prod Res. 2021:1–18.

[22] Guo R, Lee HL, Swinney R. Responsible Sourcing in Supply Chains. Manage Sci. 2016;62(9):2722–44.

[23] Agrawal V, Lee D. The Effect of Sourcing Policies on Suppliers’ Sustainable Practices. Prod Oper Manag. 2019;28(4):767–87.

[24] Yu JJ, Tang CS, Shen ZM. Improving Consumer Welfare and Manufacturer Profit via Government Subsidy Programs: Subsidizing Consumers or Manufacturers? Manufacturing & service operations management. 2018;20(4):752–66.

[25] Yu JJ, Tang CS, Sodhi MS, Knuckles J. Optimal Subsidies for Development Supply Chains. Manufacturing & service operations management. 2020;22(6):1131–47.

[26] Saha S, Goyal SK. Supply chain coordination contracts with inventory level and retail price dependent demand. Int J Prod Econ. 2015;161:140–52.

[27] Sana SS. Preventive maintenance and optimal buffer inventory for products sold with warranty in an imperfect production system. Int J Prod Res. 2012;50(23):6763–74.

[28] Arora N, Henderson T. Embedded Premium Promotion: Why It Works and How to Make It More Effective. Marketing science (Providence, R.I.). 2007;26(4):514–31.

[29] Paul N. Bloom SHKL. How Social-Cause Marketing Affects Consumer Perceptions. Mit Sloan Manage Rev. 2006;47(2):49.

[30] Koschate-Fischer N, Stefan IV, Hoyer WD. Willingness to Pay for Cause-Related Marketing: The Impact of Donation Amount and Moderating Effects. J Marketing Res. 2012;49(6):910–27.

[31] Robinson SR, Irmak C, Jayachandran S. Choice of Cause in Cause-Related Marketing. J Marketing. 2012;76(4):126–39.

[32] Chang C. To donate or not to donate? Product characteristics and framing effects of cause-related marketing on consumer purchase behavior. Psychol Market. 2008;25(12):1089–110.

[33] Krishna A, Rajan U. Cause Marketing: Spillover Effects of Cause-Related Products in a Product Portfolio. Manage Sci. 2009;55(9):1469–85.

[34] Heyes A, Martin S. Social Labeling by Competing NGOs: A Model with Multiple Issues and Entry. Manage Sci. 2017;63(6):1800–13.

[35] Mallucci P, John G, Cui TH. Pricing Cause-Related Marketing Products. SSRN Electronic Journal. 2019(3).

[36] Xu M, Li X. The interplay between e-tailer information sharing and supplier cause marketing. Int J Prod Res. 2021:1–16.

[37] Arya A, Mittendorf B. Supply Chain Consequences of Subsidies for Corporate Social Responsibility. Prod Oper Manag. 2015;24(8):1346–57.

[38] Amaldoss W, Du J, Shin W. Media Platforms’ Content Provision Strategies and Sources of Profits. Marketing science (Providence, R.I.). 2021;40(3):527–47.

[39] Che Y. Customer Return Policies for Experience Goods. The Journal of industrial economics. 1996;44(1):17–24.

[40] Xiao T, Shi J. Consumer returns reduction and information revelation mechanism for a supply chain. Ann Oper Res. 2014;240(2):661–81.

