# Supplementary material for: Optimization and selection of cause marketing mode with the warm glow effect
Source: PLoS One. 2022 Aug 11;17(8):e0272724. doi: 10.1371/journal.pone.0272724 (PMC9371360; doi:10.1371/journal.pone.0272724)
Supplement: S1 Appendix — (DOCX) [file pone.0272724.s001.docx]

**S1 Appendix**

**Proof of Proposition 1:** Owing to $\partial^{2}\Pi_{ns}^{R}/{\partial p}_{ns}^{2}=-2<0$, let $\partial\Pi_{ns}^{R}/{\partial p}_{ns}=0$; then, we obtain $p_{ns}=(3+c+\gamma\left( \beta_{ns} \right))/4>p^{*}$. By substituting $p_{ns}$ into other functions, we also obtain $\Pi_{ns}^{R}={(1-c+2\gamma\left( \beta_{ns} \right))}^{2}/16>\Pi^{R*}$, $q_{ns}=(1-c+2\gamma\left( \beta_{ns} \right))/4>q^{*}$, and $\Pi_{ns}^{S}(\beta_{ns})=\left( 1-c \right)^{2}/8+(1-c)(2\gamma\left( \beta_{ns} \right)-2\beta_{ns}c)/8-\beta_{ns}c\gamma(\beta_{ns})/2$.

Comparing $\Pi_{ns}^{S}(\beta_{ns})$ with $\Pi^{S*}={(1-c)}^{2}/8$ in the benchmark, we know, when $H\left( \beta_{ns} \right)=(1-c)(2\gamma\left( \beta_{ns} \right)-2\beta_{ns}c)/8-\beta_{ns}c\gamma(\beta_{ns})/2$ get the maximum value, we obtain the optimal retail price $p_{ns}^{*}$ and the optimal donation ratio $\beta_{ns}^{*}$. Let $\partial\Pi_{ns}^{S}/\partial\beta_{ns}=0$; then, the two stagnation points of $\Pi_{ns}^{S}(\beta_{ns})$ are obtained: $\beta_{ns1}=(\mu+3c\mu-K)/6c\mu$, $\beta_{ns2}=(\mu+3c\mu-K)/6c\mu$, and we get $\partial\Pi_{ns}^{S}/\partial\beta_{ns}>0$ in the interval $\beta_{ns}\in[0,\beta_{ns1}]$, $\partial\Pi_{ns}^{S}/\partial\beta_{ns}<0$ in the interval $\beta_{ns}\in[\beta_{ns1},\beta_{ns2}]$. Hence, $\Pi_{ns}^{S}(\beta_{ns})$ is unimodal. When $\beta_{ns1}=(\mu+3c\mu-K)/6c\mu=\beta_{ns}^{*}$, we obtain the maximum supplier’s profit, and ${H(\beta}_{ns})>0$ in the interval $0<\beta_{ns}<1$. As we know $\beta_{ns}^{*}\in[0,1]$, we get $\mu>c$, and, due to $\beta_{ns2}>1$, there is no boundary solution.

Therefore, the equilibrium solutions of $p_{ns}^{*}$, $q_{ns}^{*}$, $d_{ns}^{*}$, $\Pi_{ns}^{R*}$,$\Pi_{ns}^{S*}$ are obtained by $\beta_{ns}^{*}$.

**Proof of Proposition 2:** This is similar to the proof of proposition 1.

Letting $\partial\Pi_{nr}^{R}/\partial p_{nr}=0$, we obtain $p_{nr}=(3+c+w_{nr}+2\gamma\left( \beta_{nr} \right)+\beta_{nr}+\beta_{nr}c)/4>p^{*}$. Thus, we get $\Pi_{nr}^{R}(\beta_{nr})=\left( 1-c+2\gamma\left( \beta_{nr} \right)-\beta_{nr}-\beta_{nr}c \right)^{2}/16$; let $\partial\Pi_{nr}^{R}/\partial\beta_{nr}=0$, then the three stagnation points of $\Pi_{nr}^{R}(\beta_{nr})$ are obtained: $\beta_{nr1}=1-(1+c)/2\mu$, $\beta_{nr2}=1-(1+c)/2\mu-\sqrt{\left( 1+c \right)^{2}-8c\mu+4\mu^{2}}/2\mu$, $\beta_{nr3}=1-(1+c)/2\mu-\sqrt{\left( 1+c \right)^{2}-8c\mu+4\mu^{2}}/2\mu$, where $\beta_{nr1}$ makes the retailer’s profit function achieve the maximum value, and $\beta_{nr2}$ and $\beta_{nr3}$ makes retailer’s profit function achieve the minimum value. Hence, the retailer’s profit function $\Pi_{nr}^{R}(\beta_{nr})$ is unimodal. As we know $\beta_{nr}^{*}=\beta_{nr1}\in[0,1]$, we get $\mu>(1+c)/2$, and no boundary solution is greater than $\Pi_{nr}^{R}(\beta_{nr}^{*})$by calculation.

Therefore, the equilibrium solutions of $p_{nr}^{*}$, $q_{nr}^{*}$, $d_{nr}^{*}$, $\Pi_{nr}^{R*}$, and $\Pi_{nr}^{S*}$ are obtained by $\beta_{nr}^{*}$.

**Proof of Proposition 4:** Similar to the proof process of proposition 2, when $\Pi_{jr}^{S}\left( \alpha\right)>\Pi_{ns}^{S*}$, we get $\alpha<\alpha_{1}$; when $\Pi_{jr}^{S}\left( \alpha\right)>\Pi^{S*}$, we get $\alpha<\alpha_{2}$; when $\Pi_{jr}^{R}\left( \alpha\right)>\Pi_{ns}^{R*}$, we get $\alpha>\alpha_{3}$. Therefore, we obtain the precondition for the retailer to implement CM in M5 as an $\alpha\in[\alpha_{3},\min\left\{ \alpha_{1},\alpha_{2} \right\}]$.

**Proof of Proposition 5:** Similar to the proof process of proposition 2, letting $\partial\Pi_{cs}^{R}/\partial p_{cs}=0$, we obtain $p_{cs}=(1+w_{cs}+\gamma\left( \beta_{cs} \right))/2$; then, letting $\partial\Pi_{cs}^{S}/\partial w_{cs}=0$, we get $w_{cs}=(1+c+\gamma\left( \beta_{cs} \right)+c\beta_{cs})/2$. Thus, we get $\Pi_{cs}^{S}(\beta_{cs})=\left( 1-c+\gamma\left( \beta_{cs} \right)-c\beta_{cs} \right)^{2}/8$; letting $\partial\Pi_{cs}^{S}/\partial\beta_{cs}=0$, three stagnation points of $\Pi_{cs}^{S}(\beta_{cs})$ are obtained: $\beta_{cs1}=1-c/\mu$, $\beta_{cs2}=(-c+\mu\sqrt{c^{2}-4c\mu+\mu(2+\mu)})/\mu$, $\beta_{cs3}=(-c+\mu\sqrt{c^{2}-4c\mu+\mu\left( 2+\mu\right)})/\mu$, where $\beta_{cs1}$ makes the retailer’s profit function achieve the maximum value, and $\beta_{cs2}$ and $\beta_{cs3}$ makes supplier’s profit function achieve the minimum value. Hence, the supplier’s profit function $\Pi_{cs}^{S}{(\beta}_{cs})$ is unimodal. As we know $\beta_{cs}^{*}=\beta_{cs1}\in[0,1]$, we get $\mu>c$, and no boundary solution is greater than $\Pi_{cs}^{S}(\beta_{cs}^{*})$ through calculation.

Therefore, the equilibrium solutions of $p_{cs}^{*}$, $q_{cs}^{*}$, $d_{cs}^{*}$, $\Pi_{cs}^{R*}$, and $\Pi_{cs}^{S*}$ are obtained by $\beta_{cs}^{*}$.

**Proof of Proposition 6:** Similar to the proof process of proposition 2, letting $\partial\Pi_{cr}^{R}/\partial p_{cr}=0$, we obtain $p_{cr}=(1+w_{cr}+\gamma\left( \beta_{cr} \right)+\beta_{cr}w_{cr})/2$. Thus, we get $\Pi_{cr}^{R}(\beta_{cr})=\left( 1-w_{cr}+\gamma\left( \beta_{cs} \right)-cw_{cr} \right)^{2}/4$, let $\partial\Pi_{cr}^{R}/\partial\beta_{cr}=0$, and three stagnation points of $\Pi_{cr}^{R}(\beta_{cr})$ are obtained: $\beta_{cr1}=1-w_{cr}/\mu$, $\beta_{cr2}=(-w_{cr}+\mu-\sqrt{w_{cr}^{2}-4w_{cr}\mu+\mu(2+\mu)})/\mu$, $\beta_{cr3}=(-w_{cr}+\mu\sqrt{w_{cr}^{2}-4w_{cr}\mu+\mu\left( 2+\mu\right)})/\mu$, where $\beta_{cr1}$ makes the retailer’s profit function achieve the maximum value, and $\beta_{cr2}$ and $\beta_{cr3}$ makes supplier’s profit function achieve the minimum value. Hence, $\Pi_{cr}^{R}(\beta_{cr})$ is unimodal. As we know $\beta_{cr}^{*}=\beta_{cr1}\in[0,1]$, we get $\mu>w_{cr}$. Letting $\partial\Pi_{cr}^{S}/\partial w_{cr}=0$, three stagnation points of $\Pi_{cr}^{S}(w_{cr})$ are obtained: $w_{cr1}$, $w_{cr2}$, and $w_{cr3}$ (expressed by implicit function), and we get $\partial\Pi_{cr}^{S}/\partial w_{cr}>0$ in the interval $w_{cr}\in$[*c,*$w_{cr1}]$, $\partial\Pi_{cr}^{S}/\partial w_{cr}<0$ in the interval $w_{cr}\in$[$w_{cr1}$*,*$w_{cr2}]$, $\partial\Pi_{cr}^{S}/\partial w_{cr}>0$ in the interval $w_{cr}\in$[$w_{cr2}$*,*$w_{cr3}]$, and $\partial\Pi_{cr}^{S}/\partial w_{cr}<0$ in the interval $w_{cr}>w_{cr2}$.

Therefore, $\Pi_{cr}^{S}(w_{cr})$ attains the maximum value at $w_{cr1}$ and $w_{cr3}$, and, in the actual solution, $w_{cr1}$ and $w_{cr3}$ are substituted into the supplier’s profit function to see the solution that makes the supplier obtain the maximum value.
